# Safety and Efficacy of the Novel OmniaSecure Defibrillation Lead Through Long-Term Follow-Up: Final Results From the LEADR Trial

**DOI:** 10.1161/CIRCEP.125.014424

**Published:** 2025-12-25

**Authors:** George H. Crossley, Prashanthan Sanders, Bert Hansky, Paolo De Filippo, Maully J. Shah, Surinder Kaur Khelae, Travis D. Richardson, Francois Philippon, John S. Zakaib, Tessa Geelen, Katherin Arias, Baerbel Maus, Pamela K. Mason, for the Lead EvaluAtion for Defibrillation and Reliability Trial Investigators, Prashanthan Sanders

**Affiliations:** 1 Vanderbilt University Medical Center, Nashville, TN (G.H.C., T.D.R.).; 2 Centre for Heart Rhythm Disorders, University of Adelaide & Royal Adelaide Hospital, South Australia, Australia (P.S.).; 3 Städtische Kliniken, Bielefeld, Germany (B.H.).; 4 ASST Papa Giovanni XXIII, Bergamo, Italy (P.D.F.).; 5 The Children’s Hospital, Philadelphia, PA (M.J.S.).; 6 Institut Jantung Negara, Kuala Lumpur, Malaysia (S.K.K.).; 7 Institut Universitaire de Cardiologie et de Pneumologie de Québec, Laval University, Canada (F.P.).; 8 Minneapolis Heart Institute Foundation, MN (J.S.Z.).; 9 Medtronic Bakken Research Center, Maastricht, the Netherlands (T.G., B.M.).; 10 Medtronic, Inc, Minneapolis, MN (K.A.).; 11 University of Virginia Medical Center, Charlottesville (P.K.M.).

**Keywords:** cardiac resynchronization therapy, catheters, defibrillator, freedom, hospitalization

## Abstract

**BACKGROUND:**

The LEADR (Lead Evaluation for Defibrillation and Reliability) trial evaluated the small-diameter (4.7F), lumenless, integrated bipolar OmniaSecure defibrillation lead. The trial exceeded primary safety and efficacy objective thresholds, demonstrating favorable efficacy at implant and a low rate of complications. Three-year term outcomes of the LEADR trial assessing the OmniaSecure lead are reported here.

**METHODS:**

The LEADR trial is a prospective, multicenter, single-arm clinical trial. Patients with an indication for de novo implantable cardioverter defibrillator/cardiac resynchronization therapy defibrillator were implanted with the OmniaSecure lead in standard right ventricle locations and followed at prespecified intervals. The lead was evaluated for safety, efficacy, and reliability through final follow-up.

**RESULTS:**

There were 643/657 patients (97.9%) successfully implanted with the OmniaSecure lead with a mean follow-up of 32.4±9.1 months (26% female, 61.9±12.9 years). Pacing capture threshold, pacing impedance, and R-wave amplitudes remained stable throughout. There was a 96.5% freedom from major study lead-related complications at 3 years. At 3 years, 22.3% of patients received appropriate therapies, that is, shock and antitachycardia pacing, with a 75.4% antitachycardia pacing efficacy. Inappropriate shock rate was 2.7% and 5.9% at 1 and 3 years, respectively.

**CONCLUSIONS:**

The final results of the LEADR trial demonstrated 3-year term safety, efficacy, and reliability of the OmniaSecure lead, emphasizing the potential utility of this lead in a wide variety of patients.

**What is Known?**

- As patients with implantable cardioverter-defibrillators are living longer and improvements are made to battery longevity, transvenous leads with high reliability and durability are more important than ever.
- The use of a lumenless, small-diameter pacing lead (SelectSecure 3830) has demonstrated proven safety, efficacy, durability, and reliability over 20 years of use.

**What the Study Adds**

- The OmniaSecure lead is built on the foundation of the SelectSecure 3830 lead with an intent for use in delivering life-saving defibrillation therapy.
- The LEADR (Lead Evaluation for Defibrillation and Reliability) clinical trial demonstrated the OmniaSecure lead has excellent 3-year safety, efficacy, and reliability using both clinical and state-of-the-art bench testing.

Implantable cardioverter-defibrillators (ICDs) can deliver therapy to convert life-threatening ventricular arrhythmias.^1^ ICDs have improved the prevention of sudden cardiac death in patients at high risk; however, their long-term performance has been limited by the transvenous lead, which has long been the weakest part of the system. Transvenous leads can cause venous occlusion, tricuspid valve regurgitation, increase infection risks, and can fail or fracture, compromising the patient’s long-term safety, usually requiring lead extraction.^2,3^

The novel OmniaSecure defibrillation lead was developed in response to some of these clinical challenges. The OmniaSecure lead is a small-diameter (4.7F) defibrillation lead built on the established lumenless SelectSecure SureScan 3830 pacing lead (Medtronic Inc, MN). The lumenless platform is a simplified design that replaces the stylet lumen with a fatigue-resistant flexible cable conductor. This reconfiguration allows for a smaller lead diameter without downsizing defibrillation lead components. The LEADR (Lead Evaluation for Defibrillation and Reliability) trial evaluated the safety and efficacy of the OmniaSecure lead, showing high defibrillation efficacy and a low rate of complications. The OmniaSecure lead is expected to be highly durable, with a projected 98.2% fracture-free rate at 10 years.^4^

The long-term outcomes of the LEADR clinical trial are presented here. The aim is to provide long-term data on the safety, efficacy, and reliability of the OmniaSecure defibrillation lead. Long-term results on the performance of the OmniaSecure lead are critical to inform clinical decisions about lead selection and device management, ensuring patient safety and effective treatment.

## Methods

### Data Availability

The data, analytic methods, and study materials are owned by the sponsor and will not be made available to other researchers for purposes of reproducing the results or replicating the procedure.

### Study Design and Patient Population

The LEADR trial was a global, prospective, multicenter, single-arm, adaptive, pivotal clinical trial (URL: https://www.clinicaltrials.gov; Unique identifier: NCT04863664).^5^ The trial was approved per local regulatory and ethics committee requirements at each of the globally participating sites. In accordance with the Declaration of Helsinki, patients with American College of Cardiology/American Heart Association/European Society of Cardiology guideline-directed indications for de novo implantation of a primary or secondary prevention ICD or cardiac resynchronization therapy defibrillator (CRT-D) were enrolled after providing written informed consent. Implanted patients were followed at 3 months, 6 months, and every 6 months thereafter until study closure through in-office and remote visits in combination with remote monitoring, when available. Independent physician committees provided trial design input, oversight, and adjudication.^6^ A Clinical Events Committee reviewed and adjudicated system and procedure-related adverse events and deaths. An Episode Review Committee reviewed and adjudicated device-treated episodes for appropriateness and effectiveness of therapy.

### Safety and Efficacy

The primary efficacy objective assessed defibrillation efficacy at implantation of the OmniaSecure defibrillation lead in the right ventricle (RV) in at least the first 95 consecutive patients completing the defibrillation testing protocol.^5^ The primary safety objective assessed freedom from study lead-related major complications at 6 months. Study lead-related major complications were prespecified as those resulting in death, hospitalization, prolongation of existing hospitalization (≥48 hours), lead fracture, and system revision (reposition, replacement, explant).^5^

Through continued follow-up, the safety, efficacy, and reliability of the OmniaSecure lead were assessed. Ongoing safety was evaluated through freedom from study lead-related major complications. Long-term efficacy was assessed using the ambulatory efficacy of appropriate therapy delivery. Reliability was assessed through fracture-free performance and electrical performance.

### Lead Description and Design

**Figure 1.**
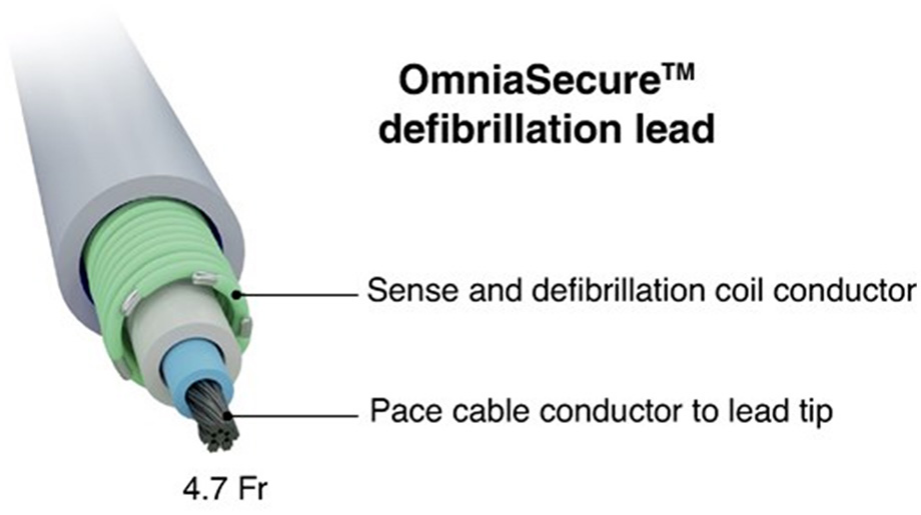
**Cross-sectional view of the OmniaSecure lead.** Adapted from Crossley et al^6^ under the Creative Commons license.

The OmniaSecure SureScan magnetic resonance imaging defibrillation lead is a small-diameter (4.7F), single-coil, integrated bipolar (6.1 cm coil length), lumenless, catheter-delivered transvenous lead (Figure 1; Medtronic Inc, Minneapolis, MN). A DF4 connector enables the OmniaSecure lead to connect to a SureScan ICD or CRT-D pulse generator (Medtronic Inc., Minneapolis, MN). In the LEADR trial, lead implantation of the OmniaSecure lead was only allowed in the standard RV position and not in conduction system locations. After fixation of the lead with the defibrillation coil outside of the catheter, electrical measurements were assessed, including pacing capture threshold, pacing impedance, and R-wave amplitude. Following implant of the study lead, implant success was defined as the connection of the device to the leads, followed by pocket closure. Physician discretion was applied to program tachyarrhythmia detection, therapies, and pacing settings.

### Statistical Analysis

Categorical variables are presented as frequencies and percentages, while continuous variables are presented using mean±SD. Ongoing safety was evaluated through Kaplan-Meier analysis for freedom from study lead-related major complications. Ambulatory efficacy was assessed as therapy (shock and ATP) efficacy using simple proportions (shock efficacy) or the generalized estimating equations method (ATP efficacy) to account for the correlation of ATP efficacy between episodes within 1 patient. Kaplan-Meier analysis was also used to determine the rates of appropriate and inappropriate therapy. CIs for Kaplan-Meier analysis were generated using log-log transformation. Reliability was evaluated by using lead electrical measurements and the fracture-free performance of the OmniaSecure lead. Descriptive analysis was used to characterize the lead electrical performance. Analysis results for the fracture-free survival rates are presented using Bayesian methodology with 95% credible intervals for the simulated patient model and clinical trial data only. Details for the clinical and simulated patient model have been previously published (see Supplemental Material for further details).^6^ Follow-up time was calculated as time from implant to exit date or last follow-up visit date if the patient was lost-to-follow-up. Device follow-up time was calculated from implant to date of last device interrogation or remote transmission. All statistical analyses were performed using SAS version 9.4 and R versions R-4.4.1 and R-4.4.2.

## Results

### Patients and Follow-Up

A total of 675 patients were enrolled in the LEADR trial across 45 global sites from June 2021 through November 2022. Of these, 657 patients underwent an implant attempt of the OmniaSecure defibrillation lead. The remaining 18 patients exited before an implant attempt. Demographics and baseline characteristics have been reported previously.^6^ These long-term results report on a mean follow-up time of 32.4±9.1 months, based on patient study visits. All subjects were followed up for a minimum of 2 years, unless they exited before the study closure for other reasons. In total, 154 patients completed 3-year follow-up visits, and 18 patients completed 3.5-year follow-up visits; the longest follow-up duration from implant to study exit was 3.9 years.

The OmniaSecure lead was successfully implanted in 643/657 (97.9%) patients (26% female, 61.9±12.9 years) with 35.9% single-chamber ICD, 40.7% dual-chamber ICD, and 23.3% CRT-D devices. The LEADR trial only allowed lead implantation in the standard RV positions, with final placements reported by physicians as low RV septum (33.1%), mid RV septum (31.9%), and RV apex (20.1%).^6^

### Efficacy

The LEADR trial passed the primary efficacy objective, demonstrating that successful defibrillation at implant exceeded the prespecified threshold of 88%.^6^ Defibrillation testing with a safety margin was conducted at implant in a subset of patients. There were 119 patients who completed the defibrillation testing protocol; of these, 116 (97.5%) were defibrillated successfully per protocol. Among the remaining 3 patients, 2 had at least a single successful conversion using 25J and remained in the study per the physician’s discretion, while the third patient received a nonstudy lead and was exited.

At the final follow-up of the LEADR trial, postimplant device data were available for episode review in all 643 successfully implanted patients, with a mean device follow-up of 30.9±9.2 months, based on device data transmissions. There have been 920 ambulatory VT/VF episodes in 123 patients who received appropriate therapy (shock and ATP). Ambulatory shocks were delivered in 190 episodes (61 patients) and successfully terminated 181 (95.3%) of those through all device follow-up, with the other episodes being ATP-terminated (6), self-terminated (1), or associated with other factors (2). Three of the 6 ATP-terminated episodes were terminated by ATP during charging. The 2 episodes that were associated with other patient factors were a patient with Brugada syndrome and multiple myeloma that was previously described^6^ and a patient with an unwitnessed death (see Supplemental Material for additional information). There were 826 monomorphic VT episodes receiving ATP (standard ATP and intrinsic ATP) in 96 patients. The ATP efficacy using the generalized estimating equations model was 75.4% (95% CI, 67.9%–81.6%). There were 81 patients who had shock prevented due to ATP termination of arrhythmia.

**Figure 2.**
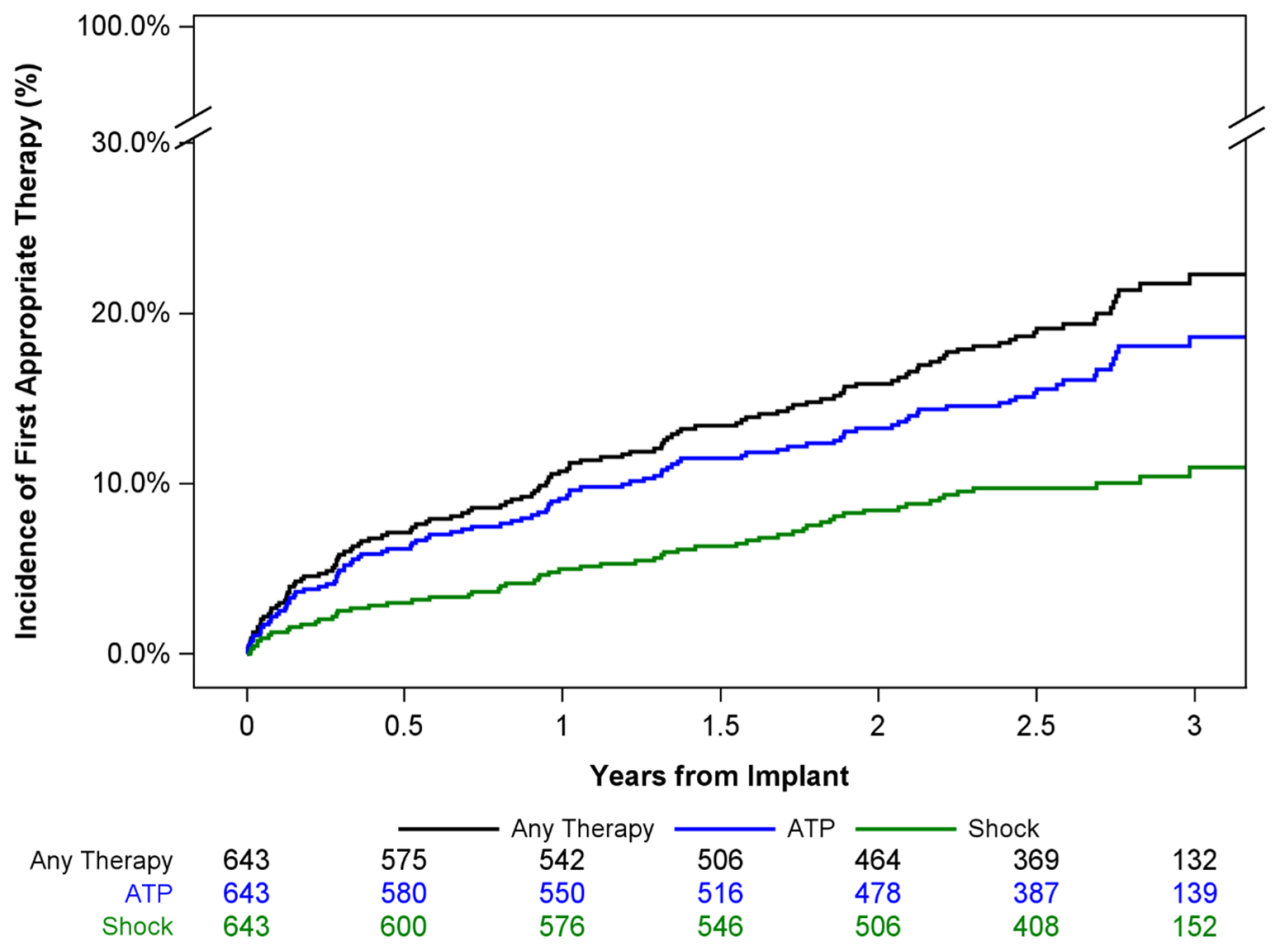
**Cumulative rate of first appropriate therapy through 3 years postimplant.** Kaplan-Meier–estimated time to first appropriate shock, ATP, or any therapy through 3 years. The number of subjects at risk is presented below in black (any therapy), blue (ATP), and green (shock).

A total of 10.7% (95% CI, 8.5%–13.4%) of patients received an appropriate therapy (shock and ATP) by 1 year and 22.3% (95% CI, 18.8%–26.3%) by 3 years, Figure 2. At 3 years, this included 11.0% (95% CI, 8.5%–14.0%) of patients receiving appropriate shock and 18.6% (95% CI, 15.4%–22.3%) receiving ATP.

Through all available device follow-up (30.9±9.2 months), there were 60 episodes with inappropriate shocks in 32 patients. The inappropriate shock rate for patients was 2.7% at 1 year (95% CI, 1.7%–4.3%) and 5.9% at 3 years (95% CI, 4.2%–8.4%). The most common reasons for inappropriate shock were as follows: AF/Atrial flutter (38 episodes in 16 patients), other SVT/sinus tachycardia (9 episodes in 7 patients), and T-wave oversensing (5 episodes in 4 patients; see Table S1 for further information). Notably, there were no inappropriate shocks due to P-wave oversensing. As previously reported, there were only 4 (0.6%) adverse events related to P-wave oversensing.^7^ There were no additional adverse events related to P-wave oversensing. There were 5 (0.8%) adverse events related to T-wave oversensing; 4 were previously described,^7^ and the other is the additional inappropriate shock reported above.

### Safety

**Figure 3.**
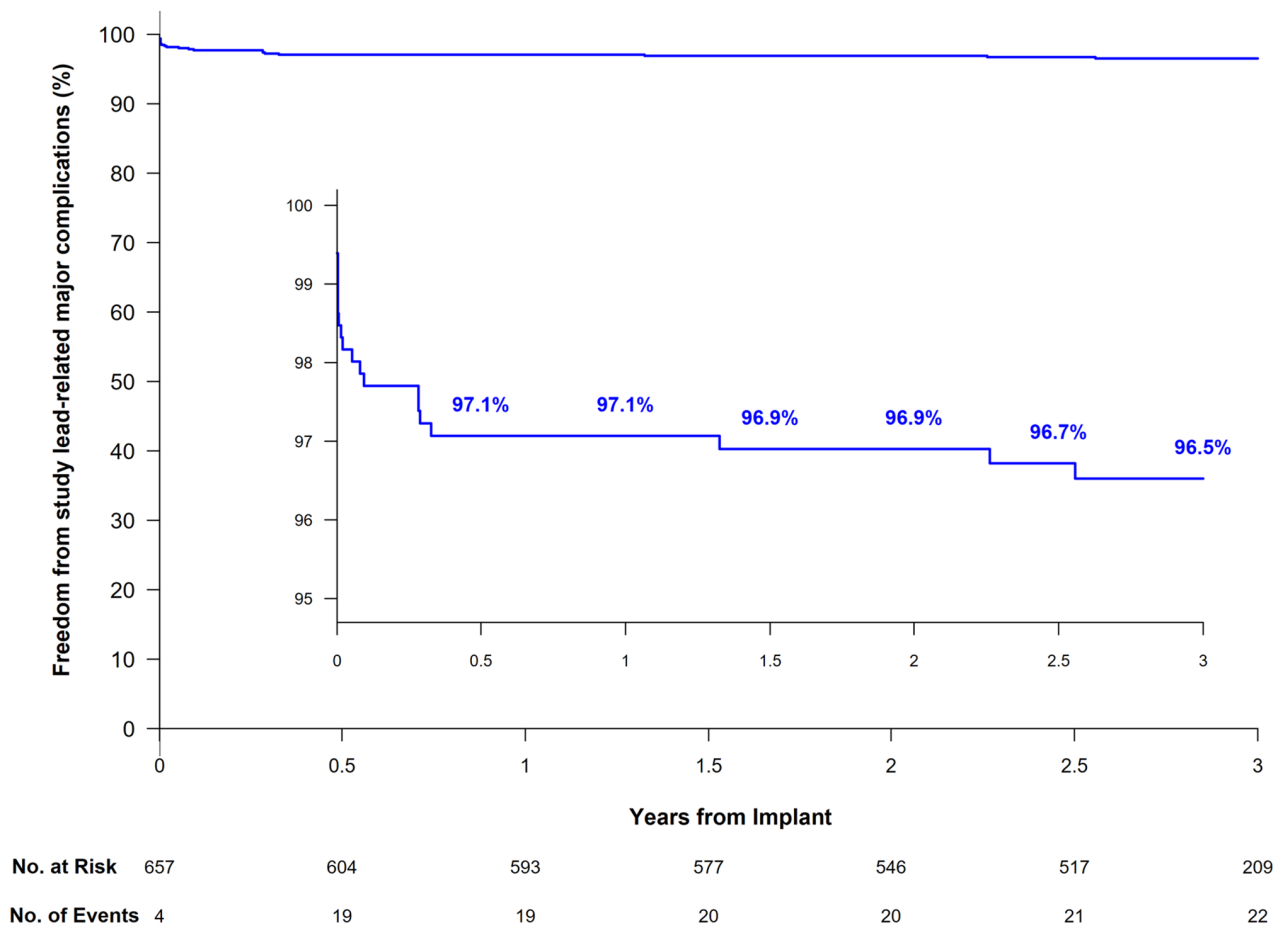
**Kaplan-Meier estimated freedom from OmniaSecure lead–related major complications through 3 years postimplant.** The estimated percentage of patients free from study lead–related major complications was 97.1% at 1 year, 96.9% at 2 years, and 96.5% at 3 years.

The LEADR trial passed the primary safety objective, demonstrating that the freedom from study lead-related major complications exceeded the prespecified threshold of 90% at 6 months.^6^ The Kaplan-Meier estimated percentage of patients free from OmniaSecure lead–related major complications was 97.1% (95% CI, 95.4%–98.1%) at 6 months and 1 year. The major complication rate changed only slightly over expanded follow-up, with a 96.5% (CI, 94.8%–97.7%) freedom from study lead-related major complications at 3 years (Figure 3). Through final follow-up, 32.4±9.1 months, there have been 22 patients with 22 study lead-related major complications, 14 of which occurred within the first 30 days postimplant. The majority of the major complications were related to lead dislodgement (N=11), followed by oversensing (N=5; Table). There was 1 study lead fracture identified through RV lead integrity alert/lead impedance alert that occurred at 2.3 years postimplant, within the predicted range of the simulated patient model (predicted fracture-free survival rate of 99.9% at 3 years), see Supplemental Material for further information. Additionally, none of the unsuccessful implants resulted in any lead-related major complications. There were no adverse events associated with the tricuspid valve that were causally related to the study lead.

**Table.**
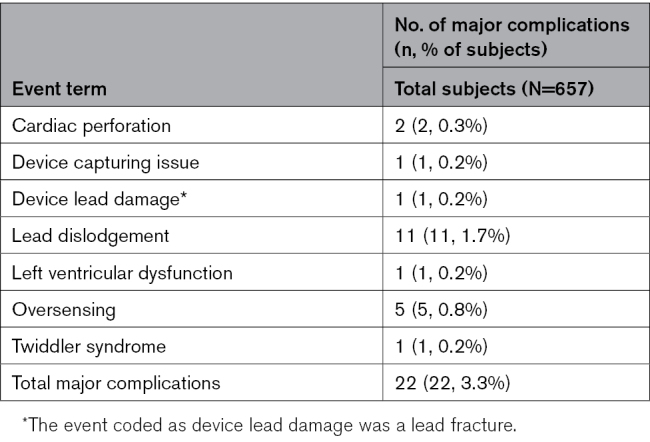
Summary of OmniaSecure Defibrillation Lead-Related Major Complications by Event Term

There was a total of 63 deaths in the LEADR trial. None of the deaths were adjudicated by the independent Clinical Events Committee as being causally related to the OmniaSecure lead. There was 1 death causally related to the ICD but not related to the OmniaSecure lead in a patient (Supplemental Material for more information).

There were 28 OmniaSecure leads explanted throughout the LEADR trial. The median dwell time was 123.5 days with a range of 0 to 777 days; there were 11 leads explanted within the first 30 days of implant. Overall, the most common reason for explant was lead dislodgement, followed by heart transplant and infection (see Table S2 for more information). There were 3 additional OmniaSecure lead explants since the previous report,^6^ of which had adverse events related to the system revision.

### Sensing and Detection Performance

**Figure 4.**
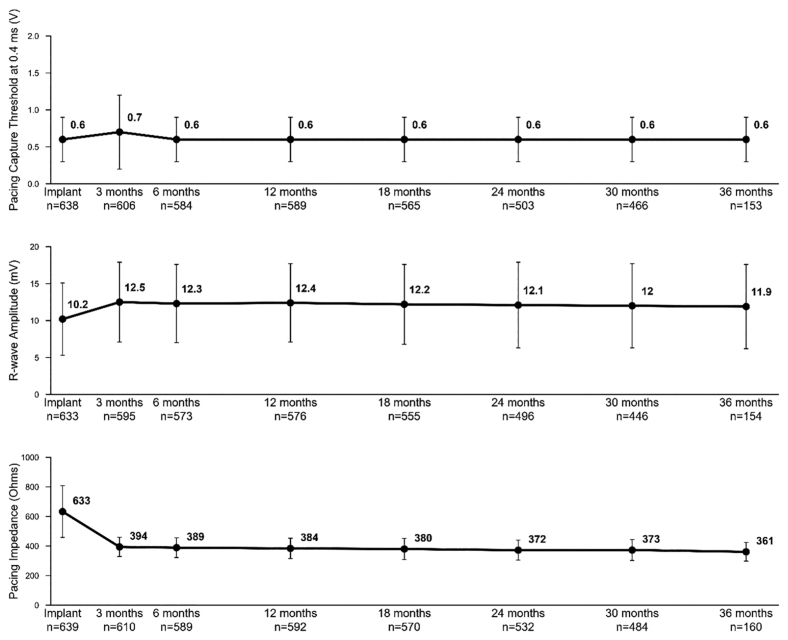
**The OmniaSecure leads electrical performance.** Pacing threshold was 0.6±0.3 V at implantation and 0.6±0.3 V, 0.6±0.3 V, and 0.6±0.3 V at 12, 24, and 36 months, respectively. R-wave amplitude was 10.2±4.9 mV at implantation and 12.4±5.3 mV, 12.1±5.8 mV, and 11.9±5.7 mV at 12, 24, and 36 months, respectively. Pacing impedance was 633±175 Ω at implantation and 384±69 Ω, 372±68 Ω, and 361±63 Ω at 12, 24, and 36 months, respectively. (All measurements used tip-to-coil polarity).

The electrical performance of the OmniaSecure lead was assessed during follow-up through mean pacing impedance, pacing capture threshold, and R-wave amplitude measurements; Figure 4. At implant, the mean pacing impedance was 633±175 Ω, 394±65 Ω at 3-month follow-up, and remained stable thereafter with 361±63 Ω at 36 months. The mean pacing capture threshold was 0.6±0.3 V at implant and remained stable thereafter, with a mean pacing capture threshold at 36 months of 0.6±0.3 V. At implant, the mean R-wave amplitude was 10.2±4.9 mV, 12.5±5.4 mV at 3-month follow-up, and remained stable thereafter with 11.9±5.7 mV at 36 months.

## Discussion

The long-term outcomes of the LEADR trial confirm that the OmniaSecure defibrillation lead performs well and is reliable through 3-year follow-up. These results show sustained safety and efficacy of the OmniaSecure lead and align with previously published analyses.^6,7^ The primary efficacy and safety objectives of the LEADR trial were met, and the prespecified study thresholds were exceeded. Across the extended 3-year follow-up, the lead had a low major complication rate of 3.5% (96.5% freedom rate), high implant defibrillation efficacy (97.5%), a high rate of appropriate ATP and shock therapy delivery (22.3%), and high reliability with stable electrical parameters.

Over the past 10 to 20 years, ICD/CRT-D device longevity has increased, such that single-chamber ICDs are projected to last over 12 years.^8^ Patients receiving these devices are also living longer and have better options to treat heart failure. An ICD/CRT-D recipient may live for more than a decade after implant,^9^ challenging even highly reliable existing leads.^10^ Therefore, there was a need to develop even more durable leads. Previous efforts to optimize defibrillation leads focused on downsizing the lead components while keeping the design the same, leading to negative outcomes and dampening innovation in defibrillation leads over the past decade.^11,12^

The OmniaSecure defibrillation lead is unique because it is not based on downsizing a predicate defibrillation lead but rather modifying the reliable SelectSecure Model 3830 pacing lead for defibrillation applications. The SelectSecure Model 3830 pacing lead, which has been in commercial use for >20 years, is a highly reliable transvenous lead with a 97.5% survival rate at 13 years.^13^ This pacing lead is a lumenless, catheter-delivered pacing lead that has a simplified design and fewer components.^14^ Modifications of the model 3830 pacing lead for development of the OmniaSecure defibrillation lead included replacing the ring electrode with a defibrillation coil to support high voltage therapy delivery, adding insulation to the outer defibrillation coil, and increasing the thickness of the outer tubing insulation.^5^ The lumenless design and the integrated bipolar configuration allow for a smaller diameter without compromising the durability of the lead.^4,14^ Integrated bipolar leads can be associated with physiological oversensing, such as P-wave and T-wave oversensing.^15^ However, in the LEADR trial, there were only 4 patients (0.6%) with adverse events related to P-wave oversensing. There we also 5 patients (0.8%) with adverse events related to T-wave oversensing.^7^ To accommodate the integrated bipolar design, the defibrillation coil is 6.1 cm long. However, the increased size of the coil did not affect the placement of the coil, as 95.5% of implants had the entire defibrillation coil in the RV.^6^ The catheter-delivered lead design also allows for targeted placement that can be beneficial in selecting the proper location to optimize defibrillation and pacing therapy; in this trial, 99.5% of OmniaSecure leads were successfully placed in the physicians’ desired RV location.^6^

The smaller lead diameter of the OmniaSecure lead may have the potential to reduce complications such as tricuspid valve regurgitation or vascular occlusion.^16–18^ Although this trial did not have specific end points related to tricuspid valve regurgitation, the LEADR trial showed no adverse events associated with the tricuspid valve that were causally related to the study lead. Moreover, the smaller size of the lead may increase the range of patients that can benefit from it. An ad-hoc analysis showed no statistically significant differences in primary safety or efficacy end points across body mass index, height, or weight, though future evaluations with larger sample sizes will help elucidate the performance of the lead across body habitus.^19^

The OmniaSecure lead durability was demonstrated in the LEADR trial, matching the expected performance from simulated patient modeling: fracture-free rates were 99.8% and 99.9% at 3 years within the clinical trial and from simulated patient modeling, respectively. Furthermore, long-term reliability modeling predicts a 98.2% fracture-free performance through 10 years for the OmniaSecure lead.^4^ The OmniaSecure lead is also expected to have high durability in the younger population, with a projected 10-year fracture-free rate in adolescent pediatrics of 97.9%, beneficial to younger patients who are smaller and more active.^4^

The LEADR trial has concluded, but the OmniaSecure lead will continue to be evaluated in a long-term postapproval study (NCT07005232). The lead is also under evaluation for use in left bundle branch area locations (NCT04863664).

### Limitations

The LEADR trial was a nonrandomized, single-arm study, and therefore, a comparison or control group was lacking, which may lead to biases when interpreting results. However, the primary objectives were set with a prespecified threshold based on historical data on currently available leads to reduce this impact. Both the efficacy and safety primary objectives were met, and the prespecified thresholds were exceeded. As previously mentioned, the LEADR trial did not assess the OmniaSecure lead implanted in the conduction system pacing locations but rather the standard RV locations only. A separate trial is ongoing that assesses the OmniaSecure lead for conduction system pacing. A longer follow-up of the OmniaSecure lead would also be beneficial, extending beyond 3 years. A real-world postapproval study will continue to evaluate the safety and efficacy of the OmniaSecure lead.

### Conclusions

The long-term results of the LEADR trial demonstrated ongoing safety, efficacy, and reliability of the OmniaSecure defibrillation lead, also demonstrating strong clinical performance to effectively treat life-threatening arrhythmias in patients. The OmniaSecure lead has the potential to minimize lead-related complications due to its smaller size, which may lessen the impact on the vasculature and the tricuspid valve, leading to improved patient safety. The lead is also projected to be highly reliable due to its durable lumenless construction, increasing lead longevity and reducing the need for replacement procedures.

## Article Information

### Acknowledgments

The author would like to thank the patients for their participation in this trial as well as the participating sites (Table S3).

### Sources of Funding

This work was supported by Medtronic Inc.

### Disclosures

Dr Crossley has served as a speaker for Medtronic and Philips and as a consultant for Medtronic and Boston Scientific. Dr Sanders reports having served on the advisory boards of Medtronic, Abbott, Boston Scientific, PaceMate, and CathRx; the University of Adelaide has received on his behalf lecture and consulting fees from Medtronic, Abbott, and Boston Scientific; and the University of Adelaide has received on his behalf research funding from Medtronic, Abbott, Boston Scientific, and Becton-Dickson. Dr Hansky reports funding from Medtronic and CVRx. Dr De Filippo received consultant fees and travel support from Medtronic, Boston Scientific, Abbott, and Biotronik, as well as honoraria and advisory board participation for Medtronic and Abbott. M.J. Shah reports serving as a consultant for Medtronic and Tenaya Therapeutics. S.K. Khelae reports being a member of the speakers bureau for Bayer/Schering Pharma, Boston Scientific, Medtronic, and Pfizer; speaker fees, honoraria, consultancy, advisory board fees, investigator, and committee member for Novartis, Medtronic, Boehringer-Ingelheim, Boston Scientific, and Bayer. Dr Richardson reports serving as a consultant for Medtronic, Johnson and Johnson, and Philips; research funding from Medtronic and Abbott. Dr Philippon reports serving as a speaker for Medtronic and Boston Scientific; consultant for Medtronic and Boston Scientific; and research funding from Medtronic and Boston Scientific. Dr Zakaib reports serving as a consultant for Medtronic. Drs Geelen, Arias, and Maus report being employees of Medtronic Inc. Dr Mason reports serving as a consultant for Medtronic, Boston Scientific, and Cook, as well as honoraria from Medtronic and Cook.

### Supplemental Material

Supplemental Methods

Supplemental Results

Tables S1–S3

Figure S1

## Supplementary Material
