# Supplementary material for: Safety and Efficacy of the Novel OmniaSecure Defibrillation Lead Through Long-Term Follow-Up: Final Results From the LEADR Trial
Source: Circ Arrhythm Electrophysiol. 2025 Dec 25;19(1):e014424. doi: 10.1161/CIRCEP.125.014424 (PMC12822760; doi:10.1161/CIRCEP.125.014424)
Supplement: Supplementary file 1 [file hae-19-e014424-s001.pdf]

SUPPLEMENTAL MATERIAL

SUPPLEMENTAL METHODS

The previously described simulated patient model,[6] with results up to 2 years was updated to 4 years to be aligned with the maximum follow-up of LEADR clinical trial subjects of 3.9 years.

SUPPLEMENTAL RESULTS

There were two unsuccessful shocks that were associated with other patient factors. One episode was in a patient with Brugada syndrome and multiple myeloma that had a sudden cardiac death 47 days postimplant and was described in an earlier publication.[1] The second episode was in a patient with a recent history of COVID had an unwitnessed sudden cardiac death at 1090 days postimplant. The device delivered all available therapies (6 shocks and ATP) without termination of arrhythmia.

The inappropriate shock rate at 6 months was 1.3% (95% CI: 0.4 – 4.0%) for single chamber (N = 231), 2.2% (95% CI: 1.2 – 4.3%) for dual/triple chamber devices (N = 412), and 1.9% (95% CI: 1.1 – 3.3%) for all devices (N = 643).

The freedom from study-lead related major complications at 3 months was 97.7% (95% CI: 96.2 – 98.6%). There were 15 patients with 15 study-lead related major complications, with the majority related to lead dislodgement (N = 10).

There was one OmniaSecure lead fracture that occurred 826 days post-implant and was associated with an RV lead integrity alert and a lead impedance alert. The lead showed loss of capture. There were no inappropriate shocks due to this fracture. Radiographical images showed a sharp bend behind the device at the location of the fracture that developed due to lead and device migration postimplant and was not present on implant x-rays (**Supplemental Figure 1**). The patient underwent a system modification that showed a proximal coil fracture without insulation break. The physician decided against extraction and instead capped the lead due to left-sided superior vena cava stenosis. A new defibrillation lead and device were implanted on the right side. The patient was followed through study closure.

The fracture-free rate was evaluated by Bayesian methodology using clinical data and simulated patient data. The simulated patient data predicted a fracture-free survival rate of 99.9% (95% Bayesian credible interval of 99.4 - 100%) at 3 years. Thus, within 657 patients with implant attempt, the simulated patient data expected one lead fracture by 3 years with a possible range of 0 to 4 lead fractures by 3 years with 95% probability. This is consistent with one study lead fracture observed in the LEADR clinical trial at 2.3 years over an average follow-up of 32.4 months. The clinical data, with the addition of a non-informative Bayesian prior,[6] shows that the fracture-free survival rate at 36 months is 99.8% (95% Credible Interval: 99.3%- 99.9%).

There was a total of 63 deaths, however none were adjudicated as being causally related to the OmniaSecure lead. There was one sudden cardiac death, 1003 days postimplant, adjudicated as causally related to the ICD device. The EGM recording appeared as ventricular fibrillation with under detection without therapies delivered. The lead showed no abnormalities or malfunctions with stable lead diagnostic trends. As a result of under detection, the event was adjudicated by the independent CEC as having a causal relationship to the ICD device.

When insufficient information was available for CEC adjudication, events were conservatively adjudicated as possibly related to the system, including the lead. By this definition, there have been five deaths that were adjudicated as being possibly related to the system/lead. Three occurred within 12 months post-implant and were previously reported.[1] The remaining two deaths occurred at 1090 and 1350 days post-implant. The death at 1090 days post-implant was due to ventricular tachycardia. As described earlier, the patient with a recent history of COVID had an unwitnessed sudden cardiac death. The device delivered all available therapies (6 shocks and ATP) without termination of arrhythmia. The CEC could not determine a causal relationship from available data and thus conservatively adjudicated as possibly related to the system. The death at 1350 days postimplant was a death of unknown cause where no data was available, therefore a relationship to the lead could not be ruled out by the CEC.

Supplemental Table 1. Reasons for inappropriate shocks through the final follow-up of the LEADR trial.

Reason	Number of Episodes (Number, % of Subjects)		
	Single Chamber (N = 231)	Dual Triple Chamber (N = 412)	Successfully Implanted (N = 643)
Noise Rhythms: Non-Cardiac Oversensing	1 (1, 0.4%)	3 (3, 0.7%)	4 (4, 0.6%)
Noise Rhythms: P-Wave Oversensing	0 (0, 0.0%)	0 (0, 0.0%)	0 (0, 0.0%)
Noise Rhythms: T-Wave Oversensing	2 (2, 0.9%)	3 (2, 0.5%)	5 (4, 0.6%)
Supraventricular Rhythm: AF / Atrial Flutter	6 (5, 2.2%)	32 (11, 2.7%)	38 (16, 2.5%)
Supraventricular Rhythm: Other SVT / Sinus Tachycardia	4 (3, 1.3%)	5 (4, 1.0%)	9 (7, 1.1%)
Lead dislodgement	1 (1, 0.4%)	2 (1, 0.2%)	3 (2, 0.3%)
Cardiogenic shock death	1 (1, 0.4%)	0 (0, 0.0%)	1 (1, 0.2%)
Total	15 (13, 5.6%)	45 (19, 4.6%)	60 (32, 5.0%)

AF: Atrial Fibrillation, SVT: Supraventricular tachycardia

Supplemental Table 2. Reasons for explant of the OmniaSecure defibrillation lead.

**Reason for study lead explant**	**Patients with study lead explant (N = 28)**
Decreased R-wave amplitude leading to RV oversensing	1
Elevated thresholds/lead dislodgement	2
Heart transplant	5
Infection	4
Lead dislodgement	5
Lead dislodgement & Routine/Prophylactic/Elective replacement: RV Lead	1
Lead dislodgement/No capture threshold at high output	1
Lead dislodgement/TWOS	1
Loss of capture RV lead	1
Non-study lead fracture/twiddler syndrome	1
PWOS	1
PWOS and diminishing R-wave	1
Pericardial effusion	1
Pocket infection	1
Pocket infection/Pocket Incision Dehiscence	1
Unusual sensing parameters	1

Supplemental Table 3. List of principal study investigators from LEADR Trial sites.

Region	Center	PI Last Name	PI First Name
Australia	Ashford Hospital	Sanders	Prashanthan
Australia	Canberra Hospital	Pathak	Rajeev
Australia	Royal Adelaide Hospital	Sanders	Prashanthan
Australia	The Prince Charles Hospital	Denman	Russell Allen
Austria	Universitätsklinikum Krems	Mihalcz	Attila
Canada	Institut Universitaire de Cardiologie et de Pneumologie de Québec (IUCPQ)	Philippon	Francois
Canada	Southlake Regional Health Centre	Tsang	Bernice
Canada	University of Calgary	Sumner	Glenn
China	Jiangsu Province Hospital†	Chen	Minglong
China	Shanxi Cardiovascular Hospital	Han	Xiuebin
China	West China Hospital of Sichuan University	Liu	Xingbin
Denmark	Rigshospitalet	Philbert	Berit Thornvig
France	Centre Hospitalier Universitaire de Grenoble – Site Nord	Defaye	Pascal
France	CHU Hôpitaux de Rouen – Hôpital Charles Nicolle	Anselme	Frederic
Germany	Klinikum Bielefeld	Stellbrink	Christoph
Hong Kong	Princess Margaret Hospital	Chan	Ngai-Yin
Hong Kong	Queen Mary Hospital	Tse	Hung-Fat
Italy	Azienda Socio Sanitaria Territoriale (ASST) Papa Giovanni XXIII	De Filippo	Paolo
Japan	Kokura Memorial Hospital	Ando	Kenji
Japan	National Cerebral and Cardiovascular Center	Kusano	Kengo
Japan	Tokyo Womens Medical University Hospital	Shoda	Morio

Malaysia	Institut Jantung Negara – National Heart Institute	Khelae	Surinder Kaur
Portugal	Centro Hospitalar de Lisboa Ocidental, E.P.E.<br>Hospital de Santa Cruz	Lopes do Carmo	Pedro Miguel
Serbia	Klinicki Centar Srbije	Milasinovic	Goran
Singapore	Ng Teng Fong General Hospital	Boey	Elaine
Spain	Hospital Universitari Bellvitge	Anguera Camos	Ignasi
Spain	Hospital Universitario y Politécnico La Fe	Cano Pérez	Oscar
United Kingdom	Imperial College Healthcare NHS Trust – Hammersmith Hospital	Whinnett	Zachary
United States	Cleveland Clinic	Baranowski	Bryan
United States	Hartford Hospital	Friedman	Meir
United States	Heart Center Research	Dinerman	Jay
United States	Hospital of the University of Pennsylvania	Schaller	Robert
United States	Lehigh Valley Hospital – Cedar Crest	Bozorgnia	Babak
United States	Minneapolis Heart Institute Foundation	Zakaib	John
United States	Northwell Health South Shore University Hospital	Chinitz	Jason
United States	Presbyterian Heart Group	West	Michael
United States	Saint Lukes Mid America Heart Institute	Ramza	Brian
United States	Texas Cardiac Arrhythmia Research Foundation	Canby	Robert
United States	Texas Health Research & Education Institute	Takata	Theodore
United States	The Childrens Hospital of Philadelphia	Shah	Maully
United States	TriHealth Hatton Research Institute	Winner	Marshall
United States	University of South Florida Health (USF)	Wilson	David
United States	University of Virginia Medical Center	Mason	Pamela
United States	Vanderbilt University Medical Center	Richardson	Travis
United States	Virginia Commonwealth University Health System	Kalahasty	Gautham

United States	Virtua Lourdes Cardiology Services	Sholevar	Darius
United States	Washington University School of Medicine	Smith	Timothy

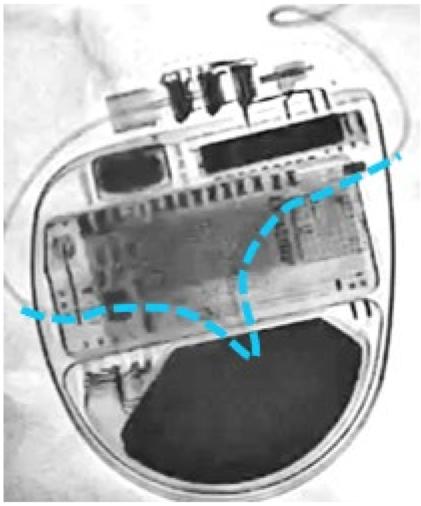

Supplemental Figure 1: Radiographical image showing a sharp bend behind the device at the location of the fracture.
